# Molecular Characterization, Phylogenetic, Expression, and Protective Immunity Analysis of OmpF, a Promising Candidate Immunogen Against *Yersinia ruckeri* Infection in Channel Catfish

**DOI:** 10.3389/fimmu.2018.02003

**Published:** 2018-09-13

**Authors:** Erlong Wang, Zhenyang Qin, Zehui Yu, Xiaohui Ai, Kaiyu Wang, Qian Yang, Tao Liu, Defang Chen, Yi Geng, Xiaoli Huang, Ping Ouyang, Weimin Lai

**Affiliations:** ^1^Department of Basic Veterinary, College of Veterinary Medicine, Sichuan Agricultural University, Chengdu, China; ^2^Yangtze River Fisheries Research Institute, Chinese Academy of Fishery Sciences, Wuhan, China; ^3^Key Laboratory of Animal Disease and Human Health of Sichuan Province, Sichuan Agricultural University, Chengdu, China; ^4^Department of Aquaculture, College of Animal Science & Technology, Sichuan Agricultural University, Chengdu, China

**Keywords:** ompF, molecular characterization, phylogenetic analysis, immunogenicity, immune effect, *Yersinia ruckeri*

## Abstract

Outer membrane porins, as the major components of Gram-negative bacterial membrane proteins, have been proven to be involved in interactions with the host immune system and potent protective antigen candidates against bacterial infection in fish. Outer membrane porin F (OmpF) is one of the major porins of *Yersinia ruckeri* (*Y. ruckeri*), the causative agent of enteric red mouth disease of salmonid and non-salmonid fish. In the present study, the molecular characterization and phylogenetic analysis of OmpF gene was studied, heterogenous expression, immunogenicity and protective immunity of OmpF were systemically evaluated as a subunit vaccine for channel catfish against *Y. ruckeri* infection. The results showed that OmpF gene was highly conserved among 15 known *Yersinia* species based on the analysis of conserved motifs, sequences alignment and phylogenetic tree, and was subjected to negative/purifying selection with global dN/dS ratios value of 0.649 throughout the evolution. Besides, OmpF was also identified to have immunogenicity by western blotting and was verified to be located on the surface of *Y. ruckeri* using cell surface staining and indirect immunofluorescence assays. Moreover, recombinant OmpF (rtOmpF) as a subunit vaccine was injected with commercial adjuvant ISA763, significantly enhanced the immune response by increasing serum antibody levels, lysozyme activity, complement C3 activity, total protein content, SOD activity, immune-related genes expression in the head kidney and spleen, and survival percent of channel catfish against *Y. ruckeri* infection. Thus, our present results not only enriched the information of molecular characterization and phylogenetics of OmpF, but also demonstrated that OmpF holds promise to be used as a potential antigen against *Y. ruckeri* infection in fish.

## Introduction

*Yersinia ruckeri* (*Y. ruckeri*) is a Gram-negative rod-shaped enterobacterium and the causative agent of enteric red mouth disease (ERM), one of the most serious septicemic bacterial disease of salmonid fish species (1). It has been reported that *Y. ruckeri* has been increasingly widespread and been detected as an important pathogen of salmonid fish in many other countries (2–6) since its isolation in North American (7–10). Apart from salmonids, *Y. ruckeri* can also infect other non-salmonid fish species including common carp (11), whitefish (12), sturgeon (13–15), and channel catfish (16, 17). Alternative approaches to traditional control strategies include probiotics and vaccines, which may play greater significance in disease control due to the increasing antibiotic resistance of bacteria (18). Although vaccines against ERM have been widely used for more than 30 years, most of these vaccines are generally inactivated whole-cell vaccines (19–22) and live-attenuated vaccines (23), which have led to selective pressure leading to emergence of other serotypes (18). Moreover, concerns about the environmental safety restricted the commercial use of such live attenuated vaccines (18). Thus, genetically engineered vaccines based on conserved and potent protective antigen genes, are increasingly urgent and need to be developed.

Outer membrane proteins (OMPs) are the major components of Gram-negative bacterial membranes and essential in maintaining the integrity and selective permeability of the outer membrane (24). As one of the membrane surface molecules, OMPs are considered as the major targets of the membrane-environment interaction and easily recognized by the infected host compared with intracellular proteins (25). Bacterial porins, one of the most abundant OMPs (26), are the main channels for many hydrophilic nutrients and antibiotics (27), and are also involved in interactions with the host immune system due to their exposed antigen epitopes on bacterial surface (28). Many studies have reported that OMPs hold promise to serve as vaccine candidate and offer significant protection against bacterial infection in fish (29–39), including OmpA (31, 32), OmpC (33), OmpK (34), OmpN (35), OmpTS (36), OmpU (37), and OmpW (38, 39). OmpF is one of the major porins of *Enterobacteriaceae*, and has been reported to be the protective antigen and to provide desirable immunoprotection against pathogenic *Escherichia coli* (40) and *Salmonella enterica* (41). Besides, based on the perspective of structure and evolution, OmpF porin gene in genus *Yersinia* was comparably conserved in structure and homology and had putative antigenic epitopes located on several loops (42), indicating that it could be used as candidate protective antigen against *Y. ruckeri* infection.

Thus, in the present study, the molecular characterization and phylogenetic analysis of *Y. ruckeri* OmpF gene was studied, heterogenous expression was conducted to serve as a candidate immunogen, the immunogenicity and protective immunity of OmpF were also systemically evaluated as a subunit vaccine against *Y. ruckeri* infection in channel catfish, which was an excellent biological model for comparative immunology research in teleosts (43–45). Based on the results of this study, OmpF gene was inferred to be a novel protective antigen of *Y. ruckeri* and recombinant OmpF (rtOmpF) was a promising vaccine candidate for channel catfish against *Y. ruckeri* infection.

## Materials and methods

### Ethics statement

The biosafety procedures of recombinant DNA technology and the use of laboratory animals in this study were carried out in strict accordance with the guidelines and recommendations of Chinese National Institute of Health. All the procedures of recombinant DNA technology and animal experiments were approved by the Institutional Animal Care and Use Committee of Sichuan Agricultural University (No. XF201418).

### Bacterial strains, plasmids, reagents, and growth conditions

*Y. ruckeri* YRWEL01, a fish pathogen isolated from dying channel catfish in Sichuan province of China, was cultured in Brain-Heart Infusion (BHI) medium at 28°C and stored at our laboratory (17). *Escherichia coli* strains DH5α and BL21 (DE3) competent cells (Takara; Dalian, China) served as cloning and protein expression host, respectively. Both strains were grown in Luria-Bertani medium containing 100 μg/ml of ampicillin (Amp) at 37°C. Plasmids pMD19-T (Takara) and pET32a (+) (Merck, Germany) served as cloning and expression vectors, respectively. Montanide™ ISA763 A VG (Seppic, France) was selected for use as an adjuvant for the experiment.

### PCR amplification and molecular cloning of OmpF

*Y. ruckeri* genomic DNA was extracted using a TIANamp Bacteria DNA extraction Kit (Tiangen, Beijing, China). The primers OmpF-F1/ OmpF-R1 of the target gene were designed using the Primer Premier 5.0 software based on the *Y. ruckeri* strain Nr34/85 OmpF gene sequence deposited in GenBank database (HM142671.1, corresponding OmpF protein accession no.: ADK27779.1). OmpF gene was amplified by PCR under the following conditions: 1 cycle of 94°C for 5 min, 30 cycles of 94°C for 1 min, 56°C for 30 s, and 72°C for 90 s, followed by a final extension of 72°C for 10 min. The product of PCR amplification was expected to be about 1098 bp and purified using the Agarose Gel DNA Extraction Kit (TaKaRa), ligated with the pMD19-T using T4 DNA ligase (TaKaRa) and transformed into *E. coli* DH5α competent cells. The positive recombinant clones were selected on the Amp/LB plate. The recombinant plasmid was identified by PCR under the aforementioned conditions, digested with restriction enzymes *Nco*I and *Sac*I, and fractionated on 1% agarose gels. DNA sequencing was conducted by TaKaRa Bio Inc. and the sequence was deposited in the NCBI GenBank to obtain accession number. The correct recombinant cloning plasmids were named as T-OmpF.

### Sequence and phylogenetic analysis of OmpF

The opening reading frame of OmpF nucleotide sequence was analyzed using ORF Finder (46). The OmpF amino acid sequences were derived from the nucleotide sequence. The signal peptide was predicted by SignalP 4.1 Server (47). To delineate the evolutionary dynamics of bacteria OmpF gene, the conserved domains and conserved motifs of OmpF in this study and other 25 reference bacteria OmpFs were searched using the Conserved Domain Database (CDD) in NCBI (48) and MEME software (49), respectively, their amino acid sequences identity were calculated using MegAlign program (DNASTAR, Madison, WI) (50), multiple sequences alignment was performed using MUSCLE (51), the phylogenetic tree was constructed using neighbor-joining method in MEGA 5 (52) with bootstrap test of 1000 replicates. To measure the selection pressures imposed on OmpF gene, the natural selection analysis was conducted based on the dN/dS ratios (the relative rates of non-synonymous (dN) and synonymous (dS) substitutions) which was calculated using Datamonkey (53).

### Cloning, expression, purification, and refolding of truncate OmpF (tOmpF)

Based on the sequence analysis, the signal peptide was removed to obtain the mature OmpF by prokaryotic expression with *E. coli* BL21 (DE3). The truncated OmpF (tOmpF) gene was amplified using primers OmpF-F2/ OmpF-R2 (Table 1), which contained *Nco*I and *Sac*I restriction enzyme sites. The resultant amplicons were purified, ligated, transformed, and sequenced as described above, and the positive recombinant cloning plasmid was named as T-tOmpF.

**Table 1:** Primers used in this study.

**Primers**	**Sequences (5′ → 3′)^a^**	**Accession no**.	**Application**
OmpF-F1	CCATGGATGAAGCGCAATATTCTTGCAGTAGTA (*Nco*I)	HM142671.1	PCR and molecular cloning
OmpF-R1	GAGCTCTTAGAACTGATAAACCAAGCCAACA (*Sac*I)		
OmpF-F2	CATGCCATGGCAGAAATCTACAACAAAG (*Nco*I)	HM142671.1	rtOmpF expression
OmpF-R2	CGAGCTCTTAGAACTGATAAACCAAGC (*Sac*I)		
18S-F	GGACACGGAAAGGATTGACAGA	AF021880.1	qRT-PCR
18S-R	GAGGAGTCTCGTTCGTTATCGG		
EF1α-F	CTGGAGATGCTGCCATTGTTG	DQ353797.1	qRT-PCR
EF1α-R	ACAGCAACGGTCTGCCTCAT		
IL-1β1-F	GCCATGTTGCTAATGTTGTAATCG	DQ160229.1	qRT-PCR
IL-1β1-R	TGTCTTGCAGGCTGTAACTCTTG		
TNF-α-F	CGCACAACAAACCAGACGAGAC	AJ417565.2	qRT-PCR
TNF-α-R	ACCACTGCATAGATACGCTCGAA		
IFN-γ-F	TGCACGAAGTGAAAGACCAAA	DQ124251.1	qRT-PCR
IFN-γ-R	TTAAGGTCCAGCAGCTCAGTGA		
CD4-L2-F	GCAGGGCACGGATAGATGGA	DQ435304.1	qRT-PCR
CD4-L2-R	TGGGTTCGCAGAGGCTGATAC		
CD8α-F	CCGACAGTGCCTACGACTAAAGC	GQ179649.1	qRT-PCR
CD8α-R	CCAGCAGCCAAAGGAATGAAG		
MHC Iα-F	GGTATCATCGTTGGTGTAGCCG	AF053547.1	qRT-PCR
MHC Iα-R	GGACAGGTTTGAAGCCAGAGTT		
MHC IIβ-F	CGGGAAGGAGATTAAAGGAGGT	U77598.1	qRT-PCR
MHC IIβ-R	GTTTGGTGAAGCTGGCGTGT		

a *Underlined nucleotides are restriction sites of the enzymes indicated in the brackets*.

The expression, purification, and refolding of recombinant tOmpF protein were performed as described in our previous studies (54, 55). Briefly, the cloned plasmid T-tOmpF was digested with *Nco*I and *Sac*I, and the resultant products and the *Nco*I /*Sac*I -digested pET32a (+) were ligated to construct the recombinant expression plasmid, named as P-tOmpF. Then, the plasmid P-tOmpF was transformed into *E. coli* BL21 and induced using 1.0 mM IPTG at 37°C for 4 h. Bacterial cells were harvested and resuspended with sterile phosphate buffer saline (PBS), followed by ultrasonication, and detection using 12.5% SDS-PAGE. The purification of recombinant tOmpF (rtOmpF), which was expressed in the form of inclusion bodies in the sediment was conducted using Ni-NTA-Sefinose Column (Sangon Biotech, Shanghai, China). The refolded tOmpF protein was obtained by gradient dialysis and analyzed using 12.5% SDS-PAGE. To rule out the potential bystander effects of LPS/or other impurities, endotoxin in recombinant protein was removed using ToxinEraserTM Endotoxin Removal kit (GenScript Corp. Nanjing, China), and the remaining endotoxin levels were measured using the Chromogenic End-point Endotoxin Assay kit (Limulus reagent biotechnology, Xiamen, China). Less than 0.1 EU/ml was detected in the final protein preparations. The protein was quantified using a NanoDrop spectrophotometer (Thermo Scientific) according to the manufacturer's instructions. Purified protein rtOmpF was stored at −20°C until further use.

### Preparation of rabbit anti-*Y. ruckeri* and anti-rtOmpF antisera

Rabbit anti-*Y. ruckeri* and anti-rtOmpF antisera were prepared according to the method described previously (56) using formaldehyde-killed *Y. ruckeri* (3.0 × 10^9^ CFU/ml) (57) and purified protein rtOmpF as the antigens, respectively. Briefly, New Zealand white rabbits were divided into three groups, one control group and two experimental groups. The purified rtOmpF (2 mg/ml) used as the antigen was emulsified with an equal volume of Freund's Complete Adjuvant (FCA, Sigma, USA) and injected intravenously into rabbits, followed by three intravenous booster injections of rtOmpF-FIA (Freund's Incomplete Adjuvant, Sigma) at 1-week intervals. Similarly, the rabbits in other two groups were immunized on the same day with PBS (control group) and formaldehyde-killed *Y. ruckeri*, respectively. After the last injection, blood was sampled from the rabbits in all three groups and centrifuged to obtain the antisera at 3,000 × g for 15 min. The antisera (Immunoglobulin G, IgG) were purified by the ammonium sulfate precipitation method (58) and stored at −20°C until required.

### Western blotting

The western blotting analysis of recombinant proteins was carried out as previously described with slight modifications (54, 55). Briefly, the purified proteins were separated using 12.5% SDS-PAGE and transferred onto two PVDF membranes at 150 V for 2 h. The membranes were pre-blocked with TBST containing 3% Bovine Serum Albumin (BSA, Sangon Biotech) for 1 h at 37°C, then incubated with rabbit anti-6 × His antibody (1:1000, Sangon Biotech) and rabbit anti-*Y. ruckeri* antisera (1:200) respectively for 12 h at 4°C. After washing three times with TBST, the membranes were incubated with goat-anti-rabbit IgG-HRP (1:5000, Sangon Biotech) for 1h at 37°C. The reaction was visualized using DAB (Sigma) for 5 to 15 min, and terminated by rinsing with distilled water.

To verify the cross-protection of OmpF in *Yersiniaceae* species, *Y. ruckeri* YRWEL01 (used in this study), *Yersinia enterocolitica*, and *Yersinia pestis* were cultured overnight and homogenized as protein sources for Western blotting. They were incubated with rabbit anti-rtOmpF sera.

### Surface display of *Y. ruckeri* OmpF

#### Cell surface staining of bacteria

The OmpF protein on the surface of *Y. ruckeri* was detected and verified using the cell surface staining method described previously (56). Briefly, *Y. ruckeri* was distributed uniformly on poly-L-lysine-treated slides (Boster, Wuhan, China) after culturing overnight on BHI agar plates. After air drying, flame fixation, and fixation in 100% methanol for 10 min at −20°C, *Y. ruckeri* coated on the slides were incubated with rabbit anti-rtOmpF sera (1:200), anti-*Y. ruckeri* sera (positive control), and rabbit negative sera (PBS group, negative control) respectively for 1 h at 37°C. After washing three times with PBST, goat anti-rabbit IgG-HRP (1:5000) was applied and incubated for 1 h at 37°C. The reaction wase visualized using DAB for 5 to 15 min and stopped by rinsing with distilled water. Then the slides were covered by cover glasses and observed using a Nikon microscope (Japan) at a × 1000 magnification.

#### Indirect immunofluorescence

To verify the surface localization of OmpF protein on *Y. ruckeri*, indirect immunofluorescence assay was also carried out as described previously with minor modification (59). Briefly, *Y. ruckeri* was distributed uniformly on poly-L-lysine-treated slides (Boster, Wuhan, China) after culturing overnight on BHI agar plates. After air drying, flame fixation, and fixation in 100% methanol for 10 min at −20°C, *Y. ruckeri* coated on the slides were incubated with rabbit anti-rtOmpF sera (1:200), anti-*Y. ruckeri* sera (positive control), and rabbit negative sera (negative control) respectively for 1 h at 37°C. After washing three times with PBS, each slide was incubated with fluorescein isothiocyanate (FITC) conjugated goat anti rabbit IgG-FITC (1:64, Sangon Biotech) for 1 h at 37°C. The reaction was stopped by rinsing with PBS and cover glasses were covered in a solution containing glycerol and PBS (1:1). The slides were observed using a fluorescence microscope (Nikon Eclipse 80i).

### Preparation of fish and vaccines

Channel catfish (50.0 ± 5.0 g) were purchased from a fish farm in Chengdu (Sichuan, China) and acclimatized in the laboratory for 2 weeks at 28 ± 1°C before any experimental manipulation. Fish were fed a commercial diet daily and water was partially replaced every day. Before the experiments, blood and tissues including liver, kidney, and spleen were sampled to detect the bacteria. No bacteria was recovered and agglutination tests showed no reaction between the serum and *Y. ruckeri*. Fish were anesthetized using MS-222 (Sigma) before any experimental manipulation. The recombinant antigen rtOmpF was diluted in PBS to obtain appropriate concentration. To obtain PBS+ ISA763 and rtOmpF+ISA763, the PBS and rtOmpF were emulsified respectively with commercial adjuvant Montanide™ ISA763 at a ratio of 3:7 by ultrasonic disruption (JY92-IIDN, Ningbo Scientz, China). The effective concentration of rtOmpF injected into fish was set as 1.0 mg/ml, which was determined using BCA Protein Assay Kit (Nanjing Jiancheng Bioengineering Institute, Nanjing, China), according to the manufacturer's description.

### Vaccination and bacterial challenge

Healthy channel catfish were divided randomly into four groups (100 fish /group) including one control group and three test groups. The vaccination protocol was carried out as described previously with some modification (55). Briefly, fish were injected intraperitoneally (i.p.) with 0.2 ml of PBS (control group), PBS+ ISA763, rtOmpF, and rtOmpF+ISA763 respectively, i.e., effective dose of rtOmpF was 4 μg/g fish, which was determined to be an optimal dose in our preliminary experiments. To obtain an optimal immune response, booster vaccination were conducted with the same method and dosage of first vaccination at 2 weeks later. At 4 weeks post-secondary vaccination (psv), 30 fish from each group were randomly selected and challenged by i.p. injection with 0.2 ml of *Y. ruckeri* YRWEL01 which was resuspended in PBS to 3.5 × 10^8^ CFU/ml (57). Mortality was monitored over a period of 14 days after the challenge, and dying fish were randomly selected for the examination of bacterial recovery from liver, kidney, and spleen. Relative percent of survival (RPS) was calculated according to the following formula: RPS = [1– (% mortality of vaccinated fish/% mortality of control fish)] × 100 (60). Serum samples of five fish in each group were collected for the detection of immune related indexes at 1-8 week psv. Head-kidney and spleen tissues of five fish were taken for qRT-PCR analysis at 24 h post-challenge. Vaccination experiments were performed in duplicate.

### Enzyme-linked immunosorbent assay (ELISA)

Sera were collected from the caudal vein of vaccinated fish at 1–8 weeks psv to detect the specific antibody against rtOmpF by ELISA as described perviously (54). Briefly, rtOmpF was diluted to 50 μg/ml in a carbonate buffer (pH = 9.6). Each well of 96-well microplate was coated using 100 μL diluted rtOmpF overnight at 4°C, followed by washing three times with PBST (0.1% Tween-20 in PBS), and then blocking with 3% BSA in PBST for 2 h at 37°C. The sera were added into the wells in triplicate and subsequently incubated for 2 h at 37°C. Rabbit anti-channel catfish IgM antiserum (1:200, prepared in our laboratory) and goat-anti-rabbit IgG-HRP (1:2000) were used as primary and secondary antibodies, respectively. The reaction was visualized using the TMB kit (Tiangen, Beijing, China) and terminated with 2 M H_2_SO_4_. The absorbance was measured at 450 nm with a microplate reader (Bio-Rad, Hercules, USA).

### Measurement of innate immune parameters

The serum lysozyme activity, complement C3 activity, total protein content, and superoxide dismutase (SOD) activity were measured at 1–8 week psv to evaluate the innate immune responses using the commercial kits (lysozyme kit Cat. No: A050-01; complement C3 kit Cat. No: E032; total protein kit Cat. No: A045-3; SOD kit Cat. No: A001-01) according to the manufacturer's instructions (Nanjing Jiancheng Bioengineering Institute, Nanjing, China). The absorbance of lysozyme, complement C3, total protein, and SOD were measured at 530, 340, 562, and 550 nm, respectively, under a microtiter plate reader (Thermo, Varioskan Flash, USA).

### qRT-PCR analysis of immune-related genes expression

Head kidney and spleen were taken at 24 h post-challenge. Total RNA extraction and cDNA synthesis were performed as described in our previous study (54). qRT-PCR was carried out using SYBR® Premix Ex Taq™ II (Tli RNaseH Plus) (TaKaRa) in an ABI StepOnePlus™ System (Applied Biosystems, USA) as described previously (54). Each assay was performed in triplicate, two housekeeping genes 18S ribosomal RNA (18S) and elongation factor-1 alpha (EF1α) were used as internal control genes (reference genes). The primers used to amplify reference genes and immune-related genes were shown in Table 1. The relative expression levels of these genes were analyzed by the 2^−ΔΔ*CT*^ method with the geometric mean of the expression levels of 18S and EF1α. All data are presented as relative mRNA expression.

### Statistical analysis

Statistical analysis was performed using SPSS 19.0 software (SPSS Inc., USA). Survival data of bacterial challenge experiment were analyzed by the Kaplan-Meier methods and log-rank tests. The data differences among groups were detected using a one-way analysis of variance (ANOVA). In all cases, the significance level was defined as *P* < 0.05 and the results were presented as mean ± SE (standard error).

## Results

### Molecular characterization and sequence analysis of OmpF

**Figure 1:**
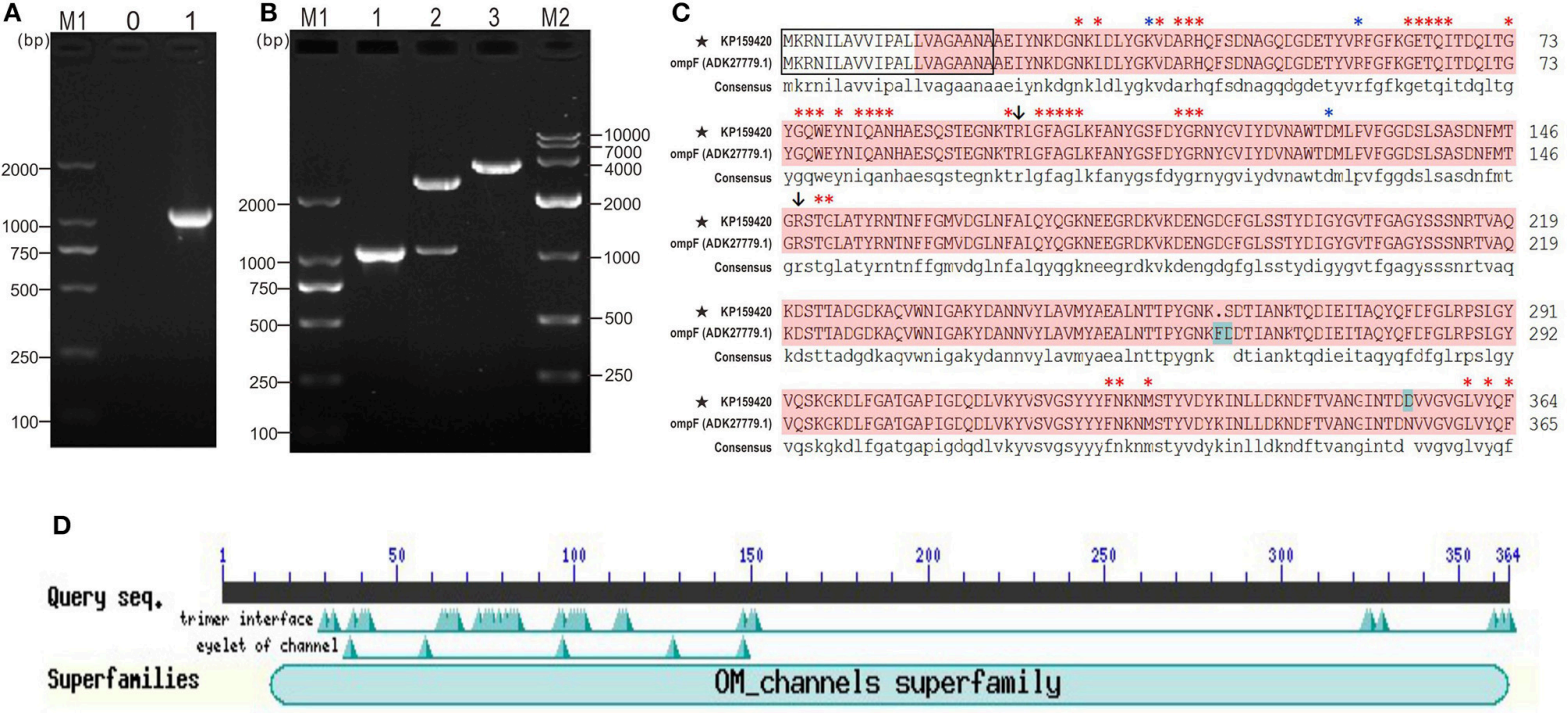
PCR, cloning, alignment and conserved domains analyses of OmpF. **(A)** PCR amplification of OmpF gene. M1: DNA marker (DL2000); lane 0: negative control; lane 1: PCR product of OmpF gene with 1095 bp. **(B)** Identification of cloning plasmid T-OmpF. M1: DNA marker (DL2000); lane 1: PCR identification of T-OmpF; lane 2: digestion of T-OmpF with NcoI and SacI; lane 3: digestion of T-OmpF with SacI [A and B are Figures 1A,B from Wang et al. (61) reproduced with permission from South China Fisheries Science]. **(C)** Amino acid sequence alignment of OmpF in this study with reference *Y. ruckeri* OmpF deposited in NCBI (ADK27779.1), black box presented the signal peptide sequences (1–22 a.a.), red shade regions presented the OM_channels superfamily conserved domain, red “^*^” indicated the trimer interface polypeptide binding sites, blue “^*^” indicated the eyelet of channel, “↓” indicated both trimer interface polypeptide binding sites and eyelet of channel. **(D)** Conserved domains of OmpF.

The coding sequence length of *Y. ruckeri* YRWEL01 OmpF gene was 1095 bp (GenBank accession No: KP159420) (Figures 1A,B), which was 3 bp shorter than reference OmpF gene (Accession No.: HM142671.1). The OmpF gene in this study contained a complete open reading frame and encoded 364 amino acid (a.a.), which was predicted to contain a 21 a.a. signal peptide (1-21 a.a.) and a 351 a.a. OM_channels superfamily conserved domain (14-364 a.a.) (Figures 1C,D). Comparing with reference OmpF amino acid sequence (Accession No.: ADK27779.1), although there were 3 a.a. differences between them, both possessed 39 trimer interface polypeptide binding sites and 5 channel eyelet sites (Figure 1C).

### Phylogenetic analysis of OmpF

**Figure 2:**
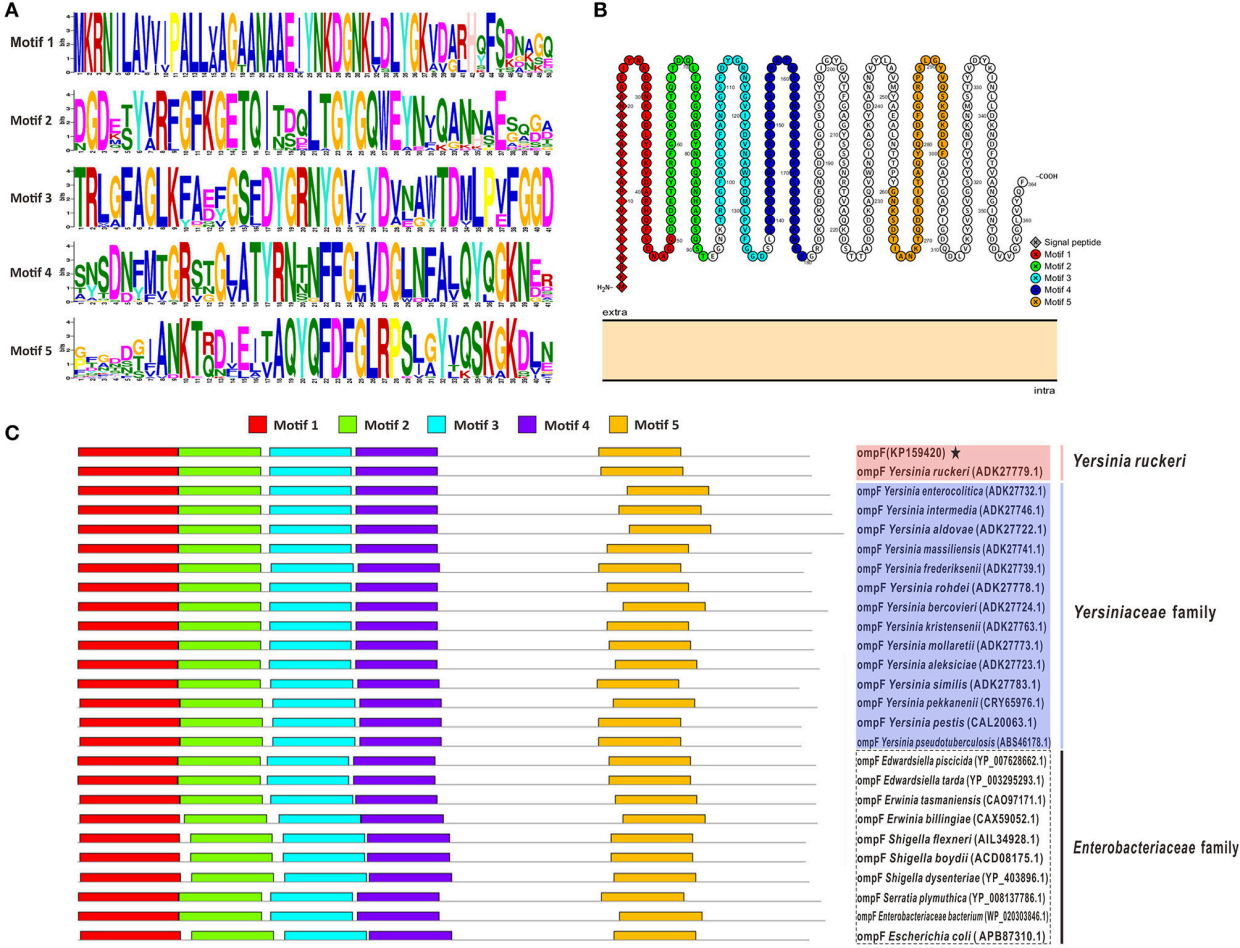
Conserved motifs analysis of OmpF with other 25 reference OmpFs. **(A)** The logos of top five conserved motifs with length ranging from five to fifty in OmpF amino acid sequences. **(B)** The distribution of these five motifs in the two-dimensional topology structures of OmpF (used OmpF in this study as an example). **(C)** The location of these five motifs in bacteria OmpFs. Red shades presented *Y. ruckeri*, blue shades presented other *Yersiniaceae* species, dotted box presented other *Enterobacteriaceae* species.

Figure 2 presented the logos of top five conserved motifs with length ranging from five to fifty (Figure 2A), as well as their corresponding locations in bacteria OmpF proteins (Figure 2C). All these reference bacteria OmpFs possessed all these five conserved motifs (Figure 2C) which constituted about 60% of the OmpF length, indicating OmpF remained comparatively conserved in different bacteria species. Besides, the two-dimensional topology structures of *Y. ruckeri* OmpF showed that OmpF located extracellular without any transmembrane regions and the signal peptide sequences located into the Motif 1 domain (Figure 2B).

**Figure 3:**
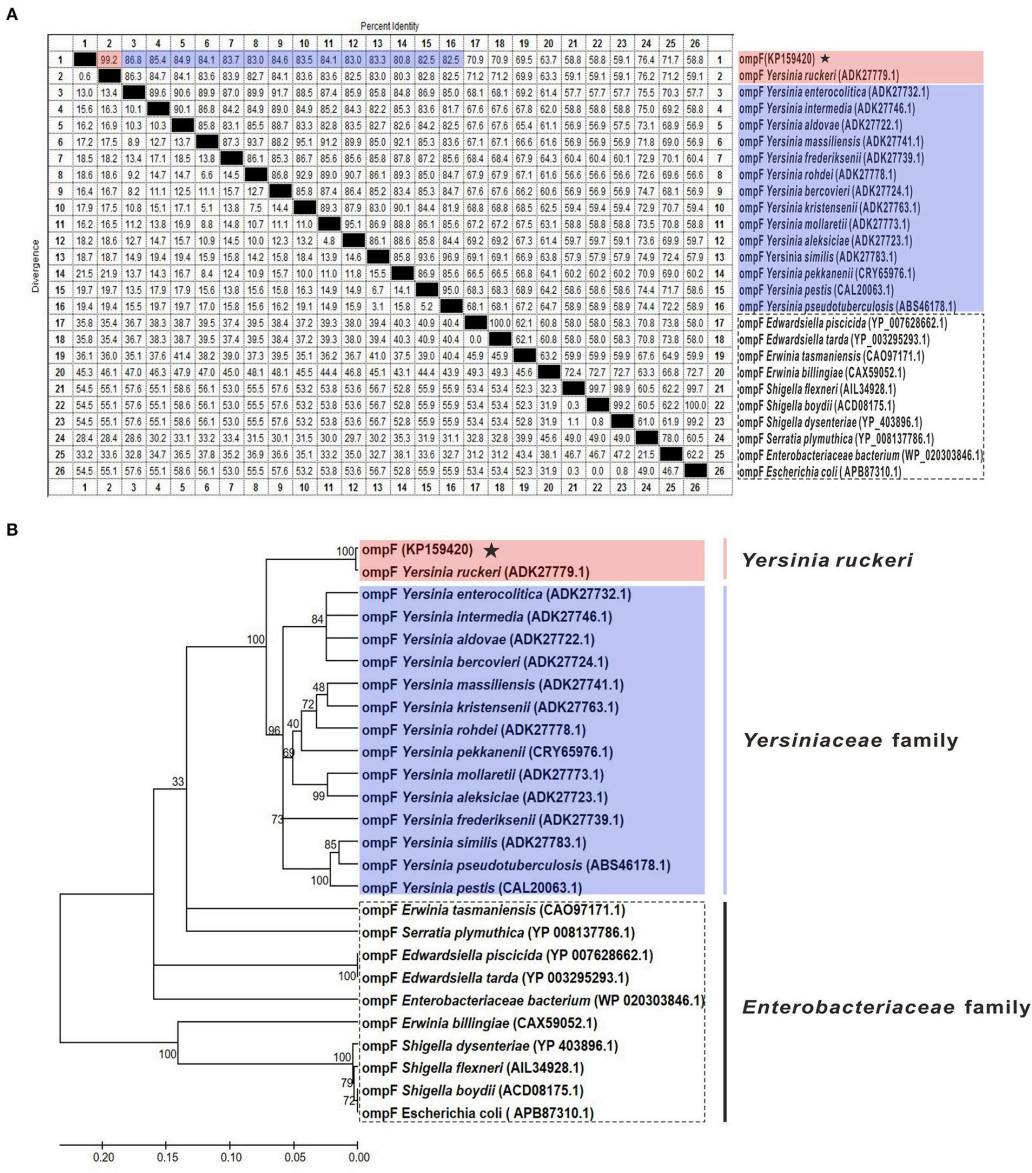
Multiple sequences alignment and phylogenetic tree of OmpF with other 25 reference OmpFs. **(A)** Multiple sequences alignment of OmpF with other 25 reference OmpFs. Red shades indicated OmpF in this study was consistent with *Y. ruckeri* OmpF with 99.2% identities, blue shades indicated other *Yersiniaceae* species OmpFs, dotted box presented other *Enterobacteriaceae* species. **(B)** Phylogenetic tree of OmpF with other 25 reference OmpFs. The numbers at each branch represent the bootstrap values obtained with 1000 replicates. “KP159420” represent the OmpF in this study.

The results of amino acid sequences identity indicated that OmpF in this study shared 99.2% identity with *Y. ruckeri* strain Nr34/85 OmpF, shared relative high identity (80.8–86.8%) with other 14 *Yersiniaceae* species OmpFs and relative low identity (58.8–76.4%) with other *Enterobacteriaceae* species OmpFs (Figure 3A). The phylogenetic analysis showed that OmpF in this study clustered one clade with *Y. ruckeri* OmpF with the bootstrap values 100, displayed a closer relationship with other bacteria of *Yersiniaceae* family and a distant relationship with other *Enterobacteriaceae* bacteria (Figure 3B). In addition, the result of natural selection pressure analysis indicated that the global dN/dS ratios of bacteria OmpFs was 0.649 with 13 positive/diversifying selection sites and 53 negative/purifying selection sites, which was well below 1.0, a theoretical boundary for positive and negative selection.

### Expression, purification and western blotting analysis of recombinant tOmpF

**Figure 4:**
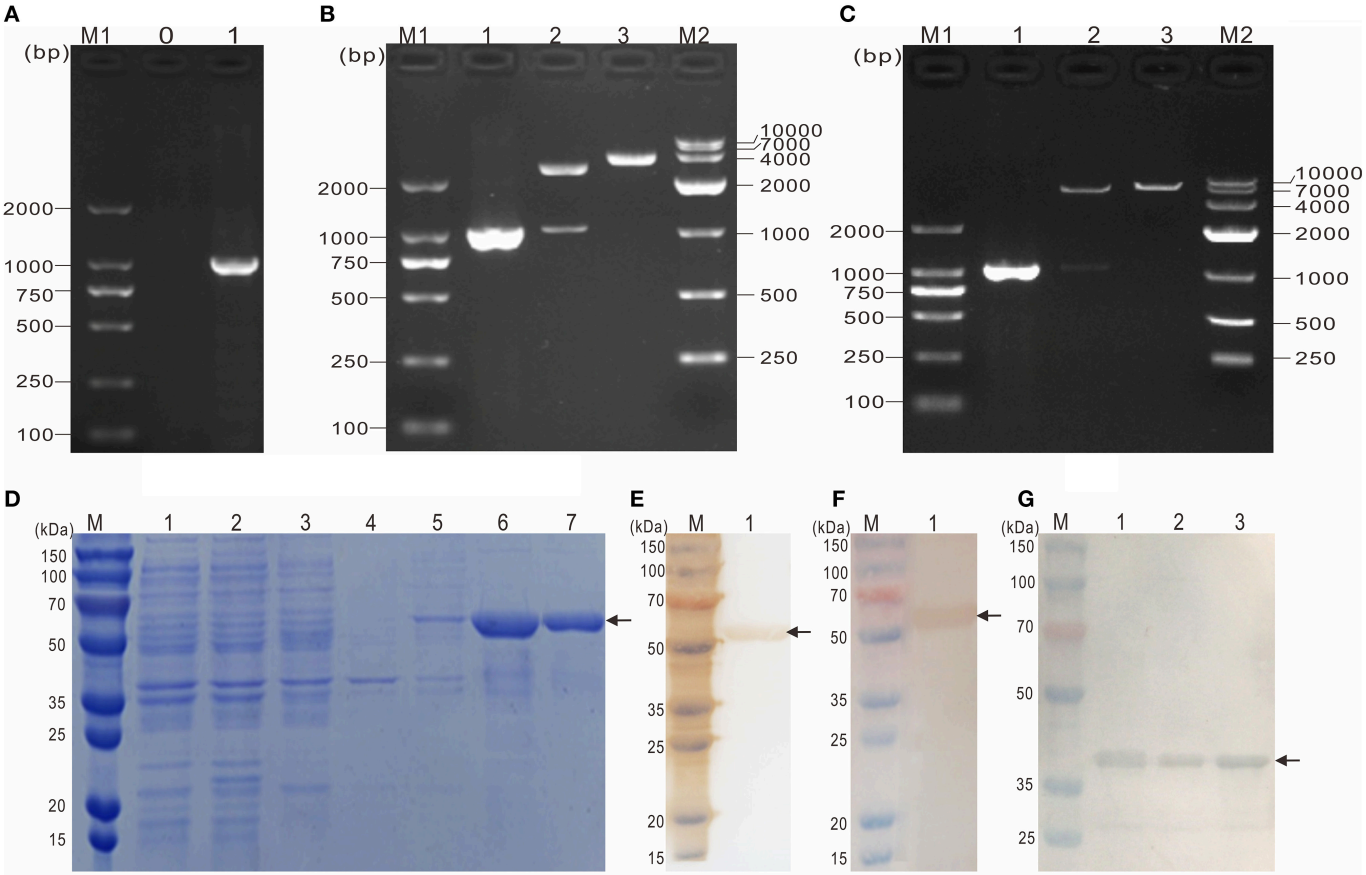
Molecular cloning, expression, purification and western blotting analysis of rtOmpF. **(A)** PCR amplification of tOmpF gene. M1: DNA Marker (DL2000); lane 0: negative control; lane 1: PCR product of tOmpF gene with 1032 bp. **(B)** Identification of recombinant cloning plasmid T-tOmpF. M1: DNA Marker (DL2000); lane 1: PCR product of T-tOmpF; lane 2: digestion of T-tOmpF with NcoI and SacI; lane 3: digestion of T-tOmpF with SacI; M2: DNA Marker (DL10000). **(C)** Identification of recombinant expression plasmid P-tOmpF. M1: DNA Marker (DL2000); lane 1: PCR product of P-tOmpF; lane 2: digestion of P-tOmpF with NcoI and SacI; lane 3: digestion of P-tOmpF with SacI; M2: DNA Marker (DL10000) [C is Figure 1C from Wang et al. (61) reproduced with permission from South China Fisheries Science]. **(D)** SDS-PAGE analysis of recombinant protein rtOmpF. M: protein marker; lane 1~6: uninduced BL21 (pET32a), induced BL21 (pET32a), uninduced BL21 (P-tOmpF) supernatant, uninduced BL21 (P-tOmpF) sediment, induced BL21 (P-tOmpF) supernatant, induced BL21 (P-tOmpF) sediment, lane 7: purified rtOmpF. **(E)** Western blotting analysis of rtOmpF with rabbit anti-6 × His antisera. M: protein marker; lane 1: specific binding between rtOmpF and rabbit anti-6 × His antisera. **(F)** Western blotting analysis of rtOmpF with rabbit anti-*Y. ruckeri* antisera. M: protein marker; lane 1: specific binding between rtOmpF and rabbit anti-*Y. ruckeri* antisera [D, E and F are Figure 10 from Wang et al. (61) reproduced with permission from South China Fisheries Science]. **(G)** The cross-protection of OmpF in *Yersiniaceae* species was analyzed by Western blotting with rabbit anti-rtOmpF sera. M: protein marker; lane 1~3: *Y. ruckeri* YRWEL01, *Y. enterocolitica* and *Y. pestis*, respectively.

To obtain the mature peptide of *Y. ruckeri* OmpF, the truncated OmpF (~1032 bp) removing the signal peptide sequence was successfully amplified using PCR and cloned into pMD19-T to obtain recombinant cloned plasmid T-tOmpF (Figures 4A, B), followed by constructing recombinant expression plasmid P-tOmpF (Figure 4C) using vector pET32a, expectedly expressed in induced *E. coli* BL21 (P-tOmpF) sediment and purifying using Ni-NTA metal affinity chromatography. After expression, purification, and refolding, recombinant proteins rtOmpF with about 55 kDa were observed using SDS-PAGE (Figure 4D). Western blotting analysis indicated that the 55 kDa band of rtOmpF specifically reacted with the rabbit anti-6 × His antisera (Figure 4E) and rabbit anti-*Y. ruckeri* antisera, respectively (Figure 4F). Furthermore, the proteins from *Y. ruckeri* YRWEL01, *Y. enterocolitica*, and *Y. pestis* were also detected by anti-rtOmpF sera with about 38 kDa (Figure 4G). Thus, we infer that rtOmpF may confer cross-protection in *Yersiniaceae* species.

### Surface display of *Y. ruckeri* OmpF

**Figure 5:**
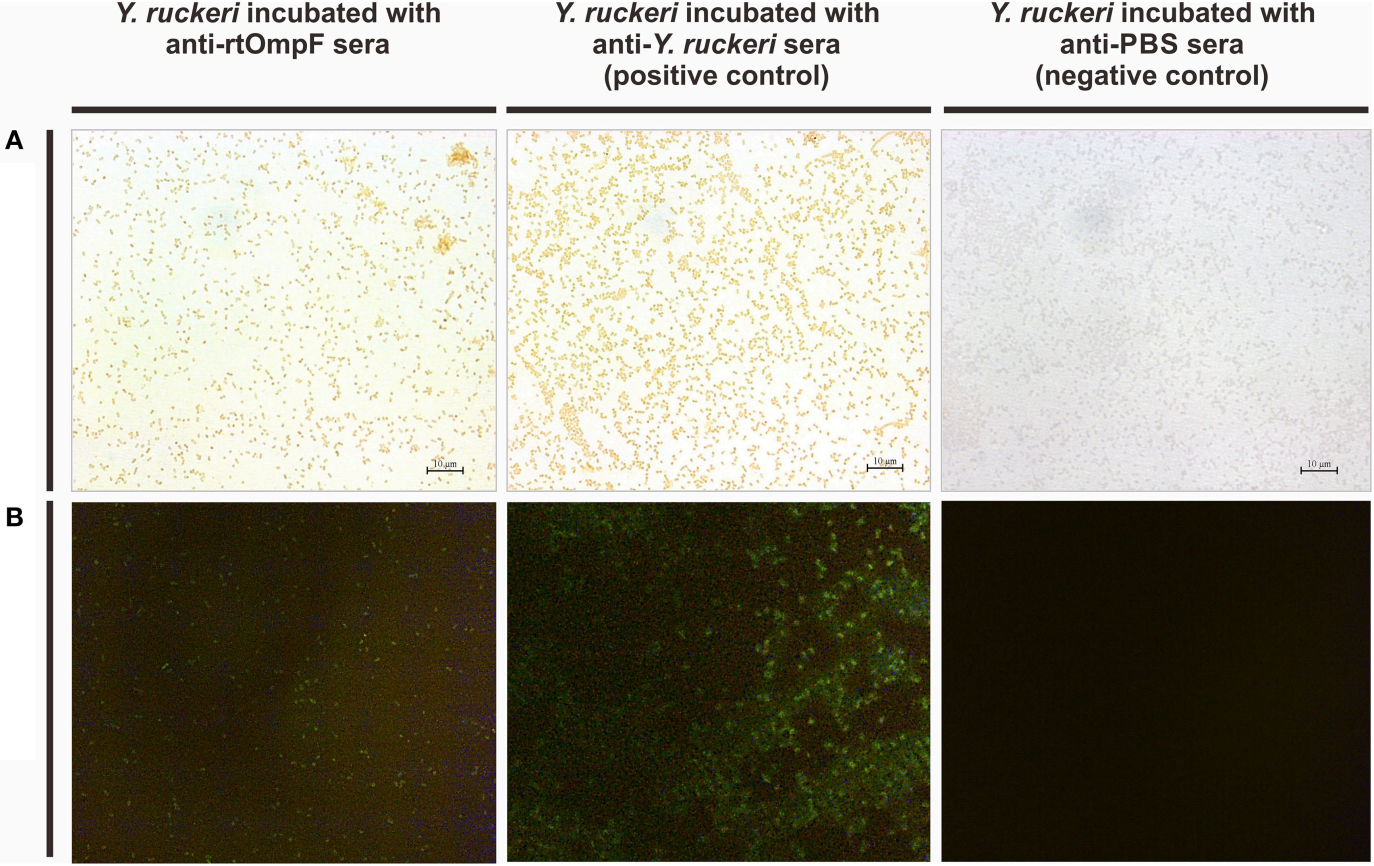
Detection of OmpF localization using bacteria cell surface staining and indirect immunofluorescence. **(A)** Cell surface staining of *Y. ruckeri*. *Y. ruckeri* incubated with rabbit negative antisera (negative control) showed no color. **(B)** Indirect immunofluorescence assay. *Y. ruckeri* incubated with rabbit negative antisera (negative control) showed no fluorescence signal.

The rabbit anti-*Y. ruckeri* antisera and rabbit negative antisera were employed as positive and negative controls, respectively. After staining with DAB, the target bacteria was recognized by rabbit anti-rtOmpF antisera and showed in brown color, which was consistent with the result of positive control, while the negative control showed no color (Figure 5A). Similarly, in indirect immunofluorescence assay, the target bacteria incubated with anti-rtOmpF antisera showed fluorescence signal, although the signal was a little weak compared with that in positive control, and the negative control showed dark without fluorescence signal (Figure 5B).

### Serum antibody production

**Figure 6:**
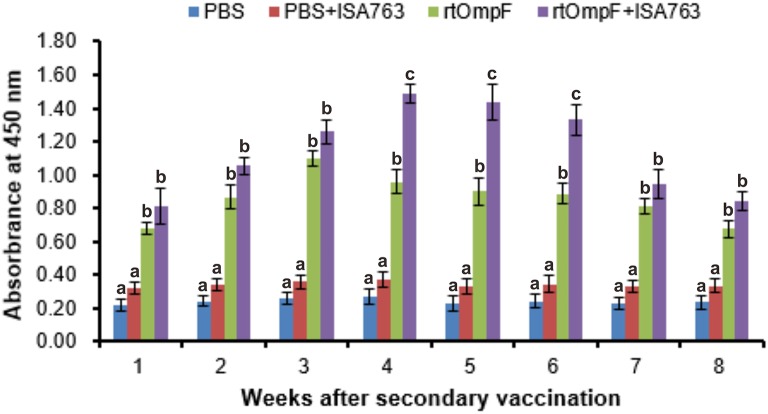
The detection of specific serum antibody in vaccinated fish using ELISA. Channel catfish were vaccinated twice at 2-week intervals, with PBS, PBS+ ISA763, rtOmpF and rtOmpF+ISA763 respectively. Sera were collected from 1st to 8th week psv. Data are presented as means ± SE (*n* = 5). Different letters above a bar denoted significant difference (*P* < 0.05).

Serum specific-antibody was detected continuously using ELISA from 1st to 8th week psv (Figure 6). The results showed that antibody levels in fish vaccinated with rtOmpF and rtOmpF+ISA763 were both significantly higher (*P* < 0.05) than that of fish vaccinated with PBS and PBS+ ISA763 at 1st−8th weeks psv. Compared with vaccine rtOmpF alone, rtOmpF+ISA763 induced significantly higher (*P* < 0.05) antibody levels at 4th, 5th and 6th weeks psv, and slightly higher antibody levels at other time points. The antibody peaks of fish vaccinated with rtOmpF and rtOmpF+ISA763 were located at 3rd and 4th week psv, respectively. During whole experimental period, the absorbance values of fish in group PBS+ ISA763 were just slight higher than that in PBS group.

### Measurement of innate immune parameters

#### Serum lysozyme activity

**Figure 7:**
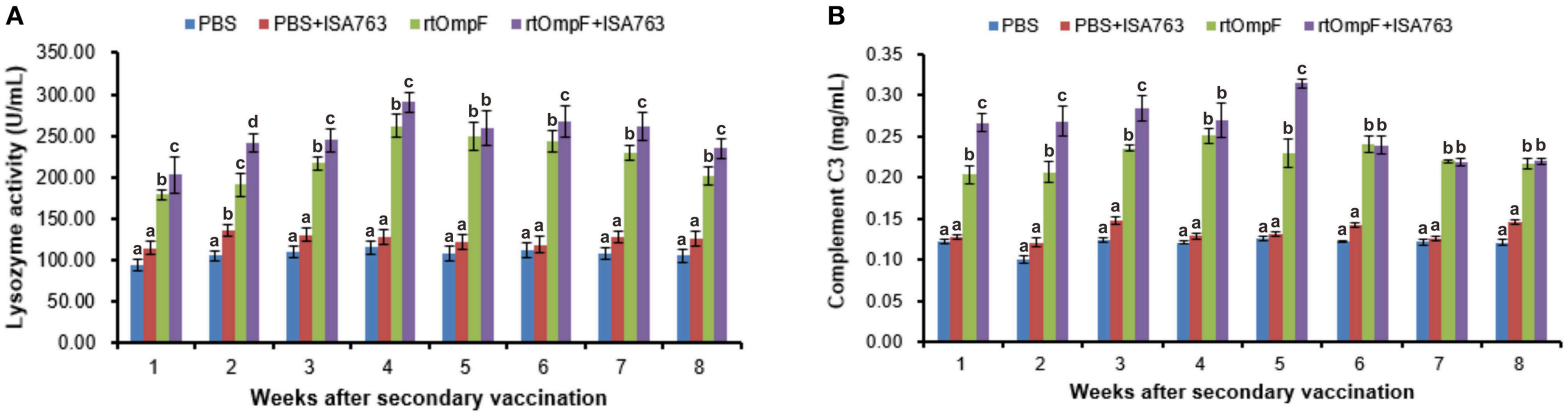
Serum lysozyme activity **(A)** and complement C3 **(B)** of vaccinated fish. Channel catfish were vaccinated twice at 2-week intervals, with PBS, PBS+ ISA763, rtOmpF and rtOmpF+ISA763 respectively. Sera were collected from 1st to 8th week psv. Data are presented as means ± SE (*n* = 5). Different letters above a bar denoted significant difference (*P* < 0.05).

The serum lysozyme activity of fish vaccinated with rtOmpF and rtOmpF+ISA763 increased significantly (*P* < 0.05) compared with that of fish in PBS and PBS+ ISA763 groups from 1st to 8th week psv (Figure 7A). During the whole experimental period, rtOmpF+ISA763 induced significantly higher (*P* < 0.05) lysozyme activity than rtOmpF alone except at 5th week psv with slight higher. The highest lysozyme activities of rtOmpF and rtOmpF+ISA763 groups were both detected at 4th week psv. Moreover, the lysozyme activity of fish in PBS+ ISA763 group was just slight higher than that in PBS group except 2nd week psv with significantly higher (*P* < 0.05).

#### Serum complement C3

Figure 7B showed the complement C3 content in serum of fish vaccinated with PBS and vaccines. The results suggested that compared with complement C3 content of fish in PBS and PBS+ ISA763 groups, the complement C3 content of fish vaccinated with rtOmpF and rtOmpF+ISA763 were significantly (*P* < 0.05) higher throughout whole detection period. Compared with rtOmpF alone, rtOmpF+ISA763 enhanced significantly higher (*P* < 0.05) complement C3 at 1st, 2nd, 3rd, and 5th weeks psv. The highest complement C3 content of fish vaccinated with rtOmpF and rtOmpF+ISA763 were detected at 4th and 5th weeks psv, respectively. Besides, the differences of complement C3 content of fish in PBS and PBS+ ISA763 were not significant from 1st to 8th week psv.

#### Serum total protein

**Figure 8:**
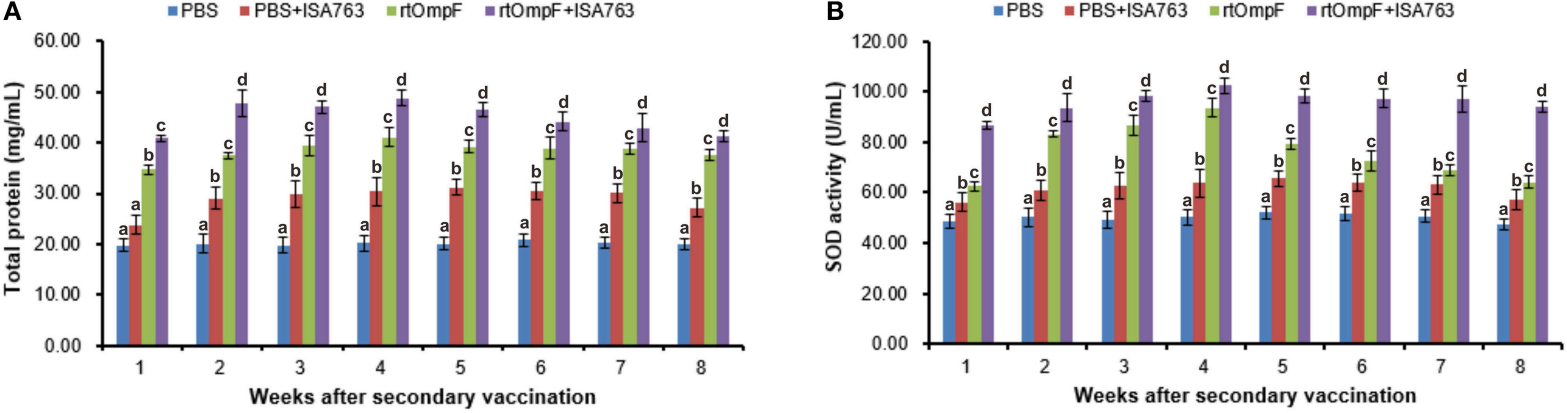
Serum total protein content **(A)** and SOD activity **(B)** of vaccinated fish. Channel catfish were vaccinated twice at 2-week intervals, with PBS, PBS+ ISA763, rtOmpF and rtOmpF+ISA763 respectively. Sera were collected from 1st to 8th week psv. Data are presented as means ± SE (*n* = 5). Different letters above a bar denoted significant difference (*P* < 0.05).

As shown in Figure 8A, the vaccines rtOmpF and rtOmpF+ISA763 both induced significantly higher (*P* < 0.05) serum total protein than PBS and PBS+ ISA763 from 1st to 8th week psv. Throughout whole detection period, the total protein levels induced by rtOmpF+ISA763 was significantly higher (*P* < 0.05) than rtOmpF alone, and the total protein levels of fish in PBS+ ISA763 group were significantly higher than that in PBS group except 1st week psv. Furthermore, the total protein peaks of fish in rtOmpF and rtOmpF+ISA763 were both observed at 4th week psv.

#### Serum SOD activity

Compared with PBS and PBS+ ISA763 groups, a significant (*P* < 0.05) increase of serum SOD activity was measured in rtOmpF and rtOmpF+ISA763 groups from 1st to 8th week psv (Figure 8B). During whole detection period, the SOD activity of fish in rtOmpF+ISA763 group was significantly (*P* < 0.05) higher than that in rtOmpF group, and the SOD activity of fish in PBS+ ISA763 group increased significantly (*P* < 0.05) compared with that in PBS group. Moreover, the highest SOD activity of vaccine groups (rtOmpF and rtOmpF+ISA763) were both detected at 4th week psv.

### Expression of the immune-related genes

**Figure 9:**
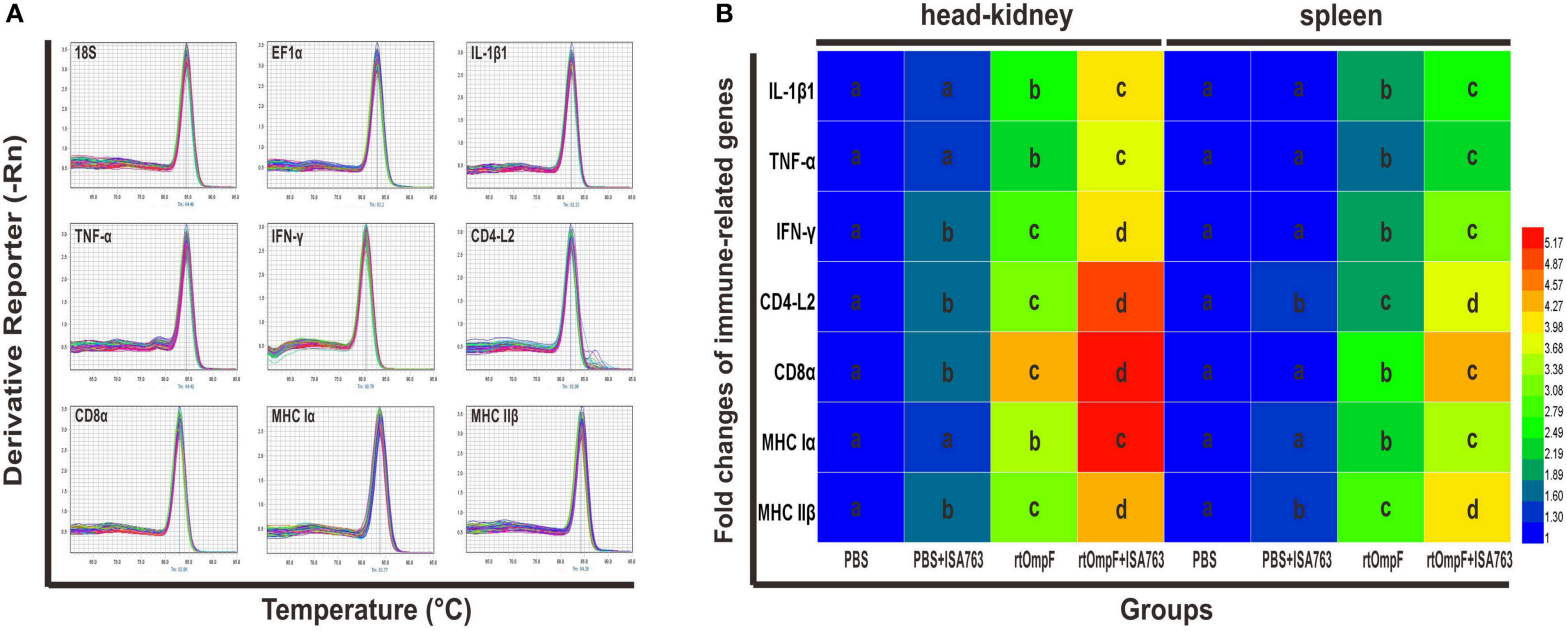
The melt curves and relative expression levels of immune-related genes in the head kidney and spleen of vaccinated fish. **(A)** The melt curves analysis of two reference genes and seven immune-related genes. **(B)** Heatmap analysis of the fold changes of immune-related genes determined by qRT-PCR in the head kidney and spleen. For each gene, the mRNA level of the PBS-vaccinated fish was set as 1. Data were presented as means (*n* = 5). Different letters in the same tissues denoted significant difference (*P* < 0.05) of the same gene in different groups. The color scale was shown at right of the figure, with blue color indicating low fold changes and red color indicating high fold changes.

The immune-related genes expression in the head kidney and spleen at 24 h post-challenge were detected by qRT-PCR with two housekeeping genes 18S and EF1α. The results of melting curve analysis indicated that there was only one peak for the PCR product of each gene (Figure 9A). The results (Figure 9B) of relative expression analysis showed that in the head kidney, the mRNA expression levels of all detected immune-related genes in rtOmpF and rtOmpF+ISA763 groups were significantly increased (*P* < 0.05) compared with those in PBS and PBS+ ISA763 groups, especially CD8α gene (more than 4.2 fold change) in rtOmpF group, CD4-L2, CD8α and MHC Iα genes (more than 4.8 fold change) in rtOmpF+ISA763 groups. Moreover, the fold changes of all these genes in rtOmpF+ISA763 group were significantly higher (*P* < 0.05) than that in rtOmpF group. In addition, similar trends were observed in spleen. Compared with PBS group, rtOmpF and rtOmpF+ISA763 induced notably higher (*P* < 0.05) relative expression of these genes, especially CD8α and MHC IIβ genes (more than 4 fold change) in rtOmpF+ISA763 group. Interestingly, the fold change of each gene in spleen was lower than that in head kidney at the same time.

### Immunoprotection efficacy against *Y. ruckeri*

**Figure 10:**
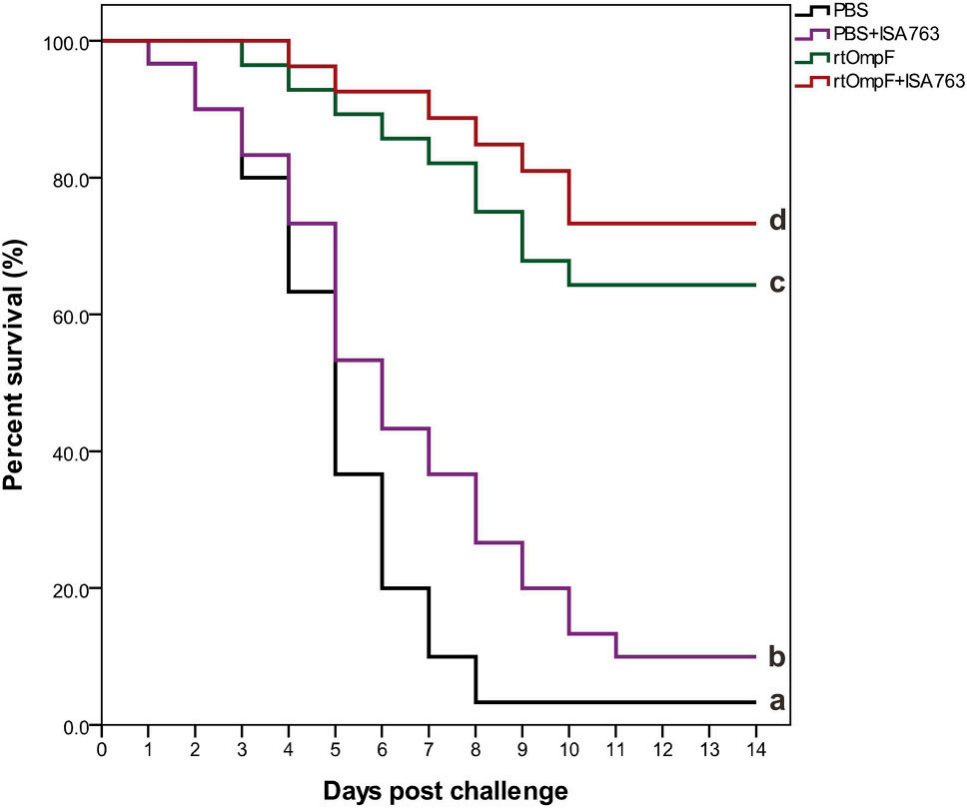
Percent survival analysis of vaccinated fish using Kaplan-Meier method. The differences among groups were analyzed by log-rank test. Different letters denoted significant difference (*P* < 0.05).

The results of survival data analyzed using the Kaplan-Meier methods indicated that the percent survivals of fish were 3.33% in PBS group, 10.0% in PBS+ISA763, 66.67% in rtOmpF group, and 76.67% in rtOmpF+ISA763 group during the challenge test with pathogenic *Y. ruckeri* YRWEL01 at 4th week psv (Figure 10). The results of log-rank analysis suggested that the survivals of fish in rtOmpF and rtOmpF+ISA763 groups were both significantly higher (*P* < 0.05) than that in PBS and PBS+ ISA763 groups. Moreover, the survival of fish vaccinated with rtOmpF+ISA763 was remarkably higher (*P* < 0.05) than that of fish vaccinated with rtOmpF alone, and the survival of fish vaccinated with PBS+ ISA763 was significantly higher (*P* < 0.05) than that of fish vaccinated with PBS alone. Besides, compared with PBS group, the immunoprotective efficacy (in terms of RPS) of PBS+ ISA763, rtOmpF and rtOmpF+ISA763 were 6.90, 65.52, 75.86%, respectively. Furthermore, *Y. ruckeri* YRWEL01 was the only type of bacterial strain detected in the liver, and kidney of moribund fish, suggesting that mortality was indeed caused by *Y. ruckeri* YRWEL01 infection.

## Discussion

With the continuous expansions of aquaculture and yield and the increasing of fish breeding density, many aquatic diseases have appeared and seriously damaged the economic productivity of aquaculture, especially bacterial diseases (62–64). Nowadays, based on the consideration of safety and antimicrobial resistance, traditional control strategies including antibiotics and chemicals are more and more questioned. By contrast, vaccines have become a more effective, safe and green intervention to control bacterial infection in aquaculture. As one of the most promising vaccines, genetically engineered vaccines such as subunit vaccines and DNA vaccines are more safe and serotype-independent due to the basis of protective immunogens (65–67). Therefore, the identification of conserved and protective immunogens is vital for the development of effective genetically engineered vaccines. As the major components and one of the most abundant proteins in the outer membrane, bacterial porin proteins play a critical role in bacterial pathogenesis and interactions with the host immune system (68, 69). OmpF, as one of the best-studied bacterial porins on structural and functional characteristics (70–74), has been reported to be a protective antigen against some bacterial infections (40, 74–77) and been predicted to be a conserved porin located on the surface of *Yersinia* (42), which suggests it is possible to use as an immunogen candidate providing immunoprotection against *Yersinia* infection.

In this study, the molecular characteristic results of *Y. ruckeri* OmpF a.a. sequence suggested OmpF was a member of OM_channels porin superfamily, a β-barrel nonspecific channels consisting of 16 antiparallel β-strands for the transportation of small hydrophillic molecules (78, 79). There were 39 trimer interface polypeptide binding sites located in OM_channels domain, which was related with the typical homotrimer structure of bacterial porins (80, 81). Conserved motifs analysis of *Y. ruckeri* OmpF with 25 reference OmpFs revealed that even though the sequence divergences existed among different bacterial OmpFs, the components and positions of conserved motifs of bacterial OmpFs remained highly conserved. Besides, multiple sequences alignment and phylogenetic analysis also indicated that OmpF remained comparatively conserved in different *Enterobacteriaceae* species, especially in *Yersiniaceae* species (sharing 80.8–86.8% identity in 14 *Yersiniaceae* species OmpFs). Moreover, the dN/dS ratio was calculated to determine the natural selection pressure imposed on OmpF gene throughout the evolution process. If a ratio dN/dS = 1, a theoretical boundary for positive/diversifying and negative/purifying selection, indicated the absence of selection. A ratio dN/dS > 1 indicated the positive/diversifying selection had occurred. By contrast, negative/purifying selection occurring on the gene should generate dN/dS <1. In the present study, the global dN/dS ratios of bacterial OmpFs was 0.649, implying that negative/purifying selection played a critical role to remove nonsynonymous substitutions from OmpF genes and OmpFs remained comparatively conserved during the evolution process and the interactions with the host immune system, which agreed with the conclusion of Stenkova et al. (42).

To verify the surface location of OmpF protein on *Y. ruckeri*, bacteria cell surface staining and indirect immunofluorescence assays were conducted with specific anti-rtOmpF antibody. Rabbit anti-*Y. ruckeri* antisera and rabbit negative antisera were employed as positive and negative controls respectively. The results suggested that positive signals were observed on the surface of *Y. ruckeri* incubated with rabbit anti-rtOmpF sera, which was consistent with the results of positive control groups, indicating that OmpF was located on the surface of *Y. ruckeri* and the polyclonal antibody against rtOmpF was successfully generated and had the desirable affinity to *Y. ruckeri*. Besides, the results of western blotting showed rtOmpF specifically reacted with the rabbit anti-6 × His antiserum and rabbit anti-*Y. ruckeri* antiserum respectively, and displayed a single predicted band with 55 kDa, which consisted of the OmpF sequence, the His-tag sequences and some sequences of expression plasmid pET32a. Furthermore, the proteins from *Y. ruckeri* YRWEL01, *Y. enterocolitica*, and *Y. pestis* can also be detected by anti-rtOmpF sera with about 38 kDa (Figure 4G), which only consisted of the OmpF sequence in strains. Taken together, these results suggested that recombinant proteins rtOmpF was expressed correctly *in vitro* and able to confer cross-protection in *Yersiniaceae* species, and harbored antigenicity properties to serve as candidate immunogen for vaccine development.

Recently, increasing studies haveshown that reference genes might not be stably expressed in different host tissues and one reference gene is not reliable enough for the accurate normalization of target genes expression (82–88). Thus, in the present study, 18S and EF1α genes, two most suitable and stably expressed genes observed in the head kidney and spleen tissues of channel catfish (89), was employed as reference genes. The geometric mean of the expression levels of 18S and EF1α was used for the accurate normalization of immune-related genes expression in qRT-PCR analysis, which was better than arithmetic mean to control the possible outlying values and abundance differences between the different genes (82). The results indicated that compared with control group, rtOmpF significantly enhanced the expression of immune-related genes involved in inflammatory response (IL-1β1, TNF-α), humoral immunity (MHC II β and CD4-L2) and cellular immunity (MHC Iα, CD8α, and IFN-γ) in the head kidney and spleen, especially co-injection of rtOmpF+ISA763, implying rtOmpF had potential as the antigen to induce a series of immune responses for channel catfish against bacterial infection, and ISA763 as the adjuvant improved the immune response. Moreover, we found that the immune-related gene expression levels in head kidney were higher than that in spleen, which may be due to their different roles and functions in fish immune response (90, 91), since head kidney served as not only the site of hematopoiesis, but also both a primary and secondary lymphoid organ, while spleen just served as a secondary peripheral lymphoid organ in fish (45).

In addition, the measurement of immune parameters including adaptive and innate immunity is the direct method to evaluate the vaccine effect. In the present study, serum specific antibody levels, lysozyme activity, complement C3 activity, total protein content, and SOD activity were detected in the control group and treatment groups. The results indicated that rtOmpF significantly enhanced the levels of above five immune parameters compared with PBS. Moreover, commercial adjuvant ISA 763 notably enhanced the immune effects induced by rtOmpF. Furthermore, survival percent and PRS were also calculated to assess the vaccine efficacy against *Y. ruckeri* infection. It was shown that the percent survival of fish in rtOmpF+ISA763 group was significantly higher than that of rtOmpF group which was remarkably higher than that of control group, and the PRS of rtOmpF and rtOmpF+ISA763 groups was 65.52 and 75.86% respectively, both were higher than that of inactive *Y. ruckeri* vaccine (RPS with 13.8%) shown in our previous study (57), mainly because of difference types, functions and mechanisms of different antigens.

In conclusion, OmpF gene was shown to be highly conserved among 15 known *Yersinia* species even in *Enterobacteriaceae* species based on the analysis of conserved motifs, sequences alignment and phylogenetic tree, and was subjected to negative/purifying selection with global dN/dS ratios value of 0.649 throughout the evolution. Besides, rtOmpF served as a candidate antigen could enhance the immune response by increasing antibody levels, lysozyme activity, complement C3 activity, total protein content, SOD activity, immune-related genes expression, and survival percent against *Y. ruckeri* infection. Moreover, co-injection of rtOmpF+ISA763 significantly improved the immune effect and immunoprotection induced by rtOmpF. Thus, our present results enriched the information of *Y. ruckeri* OmpF molecular characterization and phylogenetics, and demonstrated that OmpF holds promise to be used as a potential antigen against *Y. ruckeri* infection in fish. However, further studies are still required to understand the detailed mechanisms of OmpF used as immunogen and seek for more effective vaccine types like DNA vaccines to enhance and extend the immunoprotection.

## Author contributions

EW, ZQ, ZY, and KW designed the experiment; EW, ZQ, ZY, and XA performed the experimental work. EW, ZQ, and XA analyzed the data, EW and ZY prepared all figures, and EW drafted the paper. QY, TL, DC, and YG participated in fish vaccination and sample collection. XH, PO, and WL contributed to the discussion and revision. All authors reviewed the manuscript.

### Conflict of interest statement

The authors declare that the research was conducted in the absence of any commercial or financial relationships that could be construed as a potential conflict of interest.
